# The Concentration of Benzo[a]pyrene in Food Cooked by Air Fryer and Oven: A Comparison Study

**DOI:** 10.3390/toxics12060416

**Published:** 2024-06-06

**Authors:** Xiaoxin Chen, Yingxin Liao, Baiwen Lin, Xing He, Sibei Li, Chenghui Zhong, Saifeng Li, Yun Zhou, Lieyang Fan

**Affiliations:** School of Public Health, Guangzhou Medical University, Guangzhou 511436, China; xiaoxinchen124@163.com (X.C.); liaoyingxin2023@163.com (Y.L.); 2020163034@stu.gzhmu.edu.cn (B.L.); hx@stu.gzhmu.edu.cn (X.H.); 2020163041@stu.gzhmu.edu.cn (S.L.); zchenghui04@gmail.com (C.Z.); 18124597753@163.com (S.L.)

**Keywords:** benzo[a]pyrene (BaP), air fryer, oven, beef

## Abstract

The air fryer utilizes heated air rather than hot oil to achieve frying, eliminating the need for cooking oil, rendering it a healthier cooking method than traditional frying and baking. However, there is limited evidence supporting that the air fryer could effectively reduce the level of food-derived carcinogen. In this study, we compared the concentration of Benzo[a]pyrene (BaP), a typical carcinogen, in beef patties cooked using an air fryer and an oven, under different cooking conditions, including temperatures (140 °C, 160 °C, 180 °C, and 200 °C), times (9, 14, and 19 min), and oil added or not. The adjusted linear regression analysis revealed that the BaP concentration in beef cooked in the air fryer was 22.667 (95% CI: 15.984, 29.349) ng/kg lower than that in beef cooked in the oven. Regarding the air fryer, the BaP concentration in beef cooked without oil brushing was below the detection limit, and it was significantly lower than in beef cooked with oil brushing (*p* < 0.001). Therefore, cooking beef in the air fryer can effectively reduce BaP concentration, particularly due to the advantage of oil-free cooking, suggesting that the air fryer represents a superior option for individuals preparing meat at high temperatures.

## 1. Introduction

The air fryer is a novel cooking appliance that has emerged in recent years, which replaces hot oil with heated air to achieve the frying effect [1]. Compared with the oven, a common traditional cooking appliance, the air fryer produces fewer chemicals and carcinogens because it uses oil-free cooking. Studies have shown that the concentration of carcinogens in sturgeon cooked in the air fryer has been reduced compared with traditional deep frying [1]. The quality of cooked meats did not change significantly [2], suggesting that air fryer cooking could be a preferable choice for cooking meats. However, there is still a lack of direct evidence on whether this cooking method can effectively reduce the concentration of carcinogens in cooked meats.

Beef provides essential amino acids and vitamins necessary for the body, making it a vital component in the diet of meat consumers. Studies have shown that the moisture content on the surface of air-fried steaks decreases dramatically as the temperature increases, while the moisture content inside is maintained, resulting in higher levels of essential amino acids and the production of more flavor compounds (e.g., aldehydes, alcohols, and esters) [1,2]. However, numerous reports have shown that various polycyclic aromatic hydrocarbons (PAHs) are produced during the processing of beef, including smoking, roasting, and frying [3,4,5]. Long-term exposure to cooking-related PAHs may lead to oxidative stress in lung tissue cells, cardiovascular lesions, and cancers, among other health adverse effects [6,7,8,9]. Benzo[a]pyrene (BaP), resulting from the incomplete combustion of organic matter during the heating and roasting of fatty meat, is a prototypical carcinogen among PAHs found in food and has been classified as a Group I carcinogen by the International Agency for Research on Cancer (IARC) [10]. Studies have shown that BaP is the main carcinogen produced during beef processing [11,12,13], and the concentration of BaP in beef ham is higher than that in pork ham [14]. Apart from the meat itself, the cooking oils added during the cooking process and the cooking conditions (e.g., cooking time, cooking temperature, and cooking utensils) can influence the BaP concentration [15,16,17,18]. Due to its disruption of the human endocrine system and damage to the central nervous system, blood physiology, and DNA repair capacity [19,20], BaP has been utilized as an indicator for the presence and effects of carcinogenic PAHs in food [21]. Many countries have established limit standards for food-derived BaP: the regulatory limits of BaP concentration in smoked barbecue products and aquatic products are set at 5 μg/kg according to the National Food Safety Standard of China (GB2762-2022) [22]; in the EU, the maximum permissible limit is also set at 5 μg/kg, as stated in EU regulation 2020/1255 [23].

This study aimed to compare the differences in BaP concentration in beef patties cooked in an air fryer and a conventional oven at various temperatures, times, and with or without oil brushing. Additionally, the study identified cooking methods and conditions linked to reduced BaP concentration, thereby advocating for healthier cooking practices and promoting a nutritious diet.

## 2. Materials and Methods

### 2.1. Materials

Beef patties (wet samples, weighing 80 g, approximately 2.5 cm thick, with a diameter of 8 cm, and with a fat to lean ratio of 2:8) were transported to the laboratory in iceboxes and stored at −4 °C until cooking. Cooking oil (a blend of plant oils) was procured from the local market in Panyu District, Guangzhou City. The air fryer and oven were purchased from a local supermarket in Panyu, Guangzhou, China.

### 2.2. Sample Preparation

Based on the operating temperatures set by the manufacturer and temperatures selected for cooking beef in a previous study using an air fryer [24], a cooking temperature ranging from 140 °C to 200 °C was chosen in this work. A pre-test was subsequently conducted using the air fryer to attain a well-done beef patty under the cooking conditions of 140 °C for 19 min and 200 °C for 9 min. Therefore, we established a 5 min interval and set the cooking times at 9, 14, and 19 min. A total of 12 experimental condition sets were utilized (Table A1). The air fryer was utilized to prepare both regular beef patties and those with oil brushing, resulting in two parallel samples under each condition, totaling 72 samples. The oven was employed solely for cooking beef patties with oil brushing, generating two parallel samples for each condition, amounting to 36 samples in total. The air fryer and the oven were preheated at their respective temperatures for 2 min to ensure the desired cooking temperatures were attained.

Following the experiment, a total of 108 cooked beef patty samples were cooled, vacuum-packed, and stored at −18 °C for freezing, and then they were sent to Zhongke Testing Technology Service (Guangzhou) Co., Ltd., Guangzhou, China.

### 2.3. Determination of BaP

#### 2.3.1. Sample Pre-Treatment

The detection method and validation procedure were based on the National Standard of China, Determination of Benzo(a)pyrene in Foods: GB 5009.27-2016 [25]. The beef patties were evenly ground using a high-speed homogenizer (Blixer 3, Robot Coupe, Grasse, France), and 10 g of each sample was weighed using an analytical balance (BSA224A, Sartorius, Beijing, China, accurate to 0.001 g) and mixed with 50 mL of n-hexane. Subsequently, the mixture was vortexed for 0.5 min, ultrasonicated at 40 °C for 10 min, and centrifuged at 4000 r/min for 5 min to collect the supernatant. This extraction process was repeated by adding another 50 mL of n-hexane. The combined supernatant was then transferred into the BaP molecularly imprinted column of the solid phase extraction device (Waters, Milford, MA, USA), which was initially activated with 5 mL of dichloromethane followed by 5 mL of n-hexane. Upon reaching the column bed, the column was flushed with 6 mL of hexane, and the effluent was discarded. Subsequently, the column was eluted with 6 mL of dichloromethane, and the purified solution was collected in a test tube and dried at 40 °C under nitrogen flow using a nitrogen blowing instrument (MTN-5800, Tianjin Automatic Science Instrument Co., Ltd., Tianjin, China). Finally, the extract was dissolved in 1 mL of acetonitrile, filtered through a 0.22 µm membrane, and subjected to analysis using high-performance liquid chromatography (HPLC).

#### 2.3.2. Determination of BaP Using Tertiary Liquid Chromatography

The determination of BaP concentration was conducted using a Shimadzu liquid chromatograph LC30 (Shimadzu Corporation, Suzhou, China) equipped with a fluorescence detector. The analytes were separated on a Shim-pack Scepter C18/Shim-pack Velox C18 column (250 mm × 4.6 mm, 5 μm). The injection volume was set at 10 μL, and the mobile phases consisted of acetonitrile and water flowing at a rate of 1.0 mL/min with a column temperature maintained at 40 °C. The fluorescence detector used an excitation wavelength of 384 nm and an emission wavelength of 406 nm. BaP quantification was determined by comparing the retention time and chromatographic peak area with the standard curve of BaP. The method possesses a limit of detection of 10 ng/kg and a limit of quantification of 50 ng/kg. A validation procedure was performed to ensure the absolute difference between two independent test results obtained under conditions of reproducibility did not exceed 20 percent of the arithmetic mean. Data points below the detection limit were processed using half of the detection limit [26].

#### 2.3.3. Calculation of BaP Concentration in Samples

The formula is as follows:

$$X=\frac{\rho \times V}{m}$$

“X” represents the BaP concentration in the sample, measured in μg/kg; “ρ” denotes the concentration of the sample purification solution derived from the standard curve, measured in μg/mL; “V” stands for the final volume of the sample, measured in mL; “m” indicates the mass of the wet sample, measured in kilograms (kg).

### 2.4. Statistical Analysis

Statistical analysis was conducted using R version 4.2.3 and SPSS version 25.0. The R package “interactions” was used during statistical analysis. Prior to ANOVA analysis, normality and homogeneity of variance tests were performed on the data. In SPSS, we used the Shapiro–Wilk to test the normality of the data and Levene’s test to test the homogeneity of variances. The test results indicated that the data met the assumptions of normality and homogeneity of variances. ANOVA was employed to examine the impact of cooking utensils and oil brushing on the BaP concentration of beef patties. Spearman’s correlation analysis was utilized to investigate the relationship between cooking temperature, cooking time, and the BaP concentration of beef patties. Linear regression was employed to evaluate the influence of cooking utensils, cooking temperature, cooking time, and oil brushing on the BaP concentration in beef. Additionally, stratified analysis was conducted. A significance level of α = 0.05 and *p* < 0.05 were deemed statistically significant. Graphs were generated using GraphPad Prism version 9.

## 3. Results

**Figure 1:**
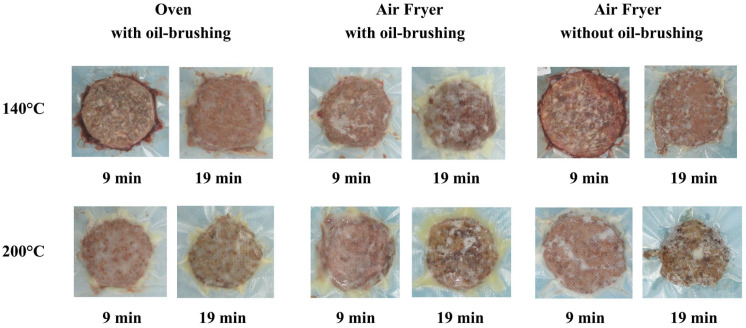
Images of beef patties after cooking.

Figure 1 displays images of beef patties after cooking under various conditions. It is observed that the beef patties retained a red color with visible traces of blood after cooking at 140 °C for 9 min, whereas the surface of the beef exhibited a charred brown appearance following cooking at 200 °C for 19 min.

Table 1 shows the BaP concentration in beef patties treated with various cooking methods. Compared with the oven, the air fryer significantly reduced the BaP concentration in beef under most cooking conditions, including 140 °C for 9 min, 160 °C for 9 min, 160 °C for 14 min, 180 °C for 14 min, 200 °C for 14 min, and 200 °C for 19 min (all *p* < 0.05). A relatively long time (19 min) at low temperature (140 °C and 160 °C) seemed to produce more BaP contained in beef when using the air fryer rather than the oven.

The impact of oil brushing on the BaP concentration was further investigated with air fryer cooking. The findings revealed that the BaP concentration in groups without oil brushing during cooking was below the detection limit and was significantly lower than that in groups with oil brushing (all *p* < 0.001).

### 3.1. Effect of Different Temperatures on BaP Concentration in Beef

**Figure 2:**
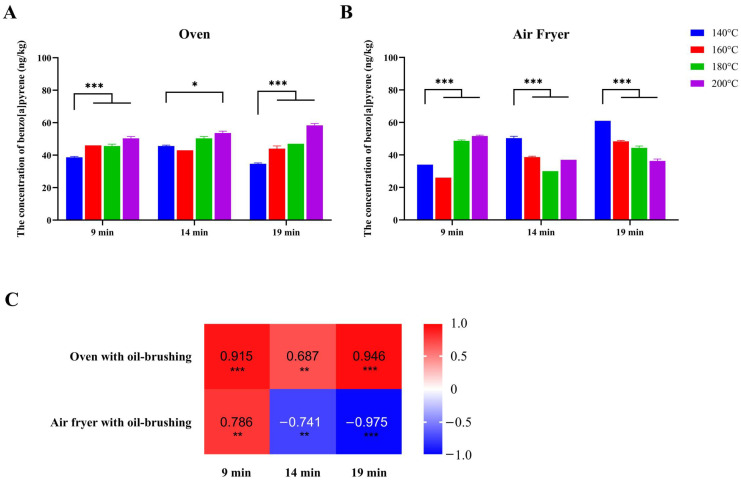
Effect of different temperatures on BaP concentration in beef. Note: Histograms denote the BaP concentrations in beef patties at different temperatures using the oven (**A**) or the air fryer (**B**). The relationships between the concentration of BaP and temperatures were evaluated using Spearman’s correlation analysis. The heatmap exhibits the rank correlation coefficients (**C**). All samples were wet samples. Asterisks indicate significant differences in BaP concentration between temperatures (* *p* < 0.05, ** *p* < 0.01, *** *p* < 0.001).

When cooking with the oven, the BaP concentration in beef cooked at 200 °C was significantly higher than that at 140 °C within each time group (all *p* < 0.05) (Figure 2A). Spearman’s correlation analysis showed that the cooking temperature and BaP concentration in beef were positively correlated within the 9 min, 14 min, and 19 min groups. The rank correlation coefficients were 0.915, 0.687, and 0.946, respectively (all *p* < 0.05) (Figure 2C).

When cooking with the air fryer, the BaP concentration in the 200 °C group was significantly higher than that in the 140 °C group when cooked for 9 min, but it was significantly lower than that in the 140 °C group when cooked for 14 min or 19 min (all *p* < 0.05) (Figure 2B). Spearman’s correlation analysis showed that cooking temperature and BaP concentration in beef were positively correlated in the 9 min group (rank correlation coefficients = 0.786), but they were negatively correlated in both the 14 min group (rank correlation coefficients = −0.741) and the 19 min group (rank correlation coefficients = −0.975) (all *p* < 0.05) (Figure 2C).

### 3.2. Effect of Different Time on BaP Concentration in Beef

**Figure 3:**
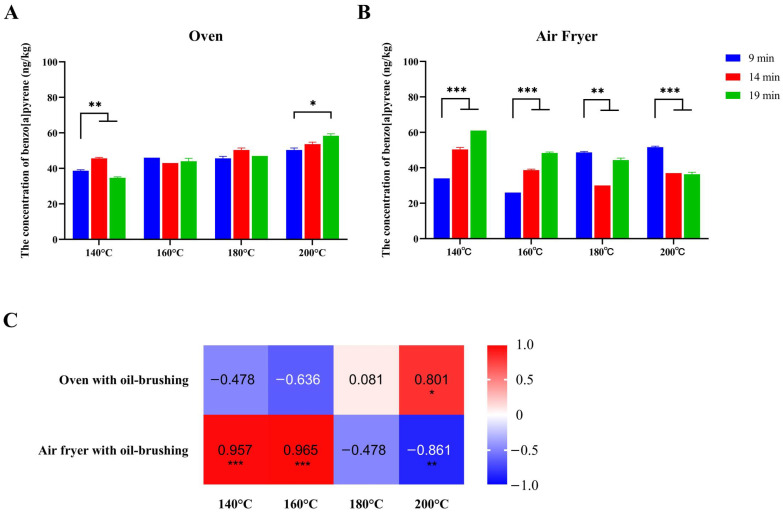
Effect of different time on BaP concentration in beef. Note: Histograms denote the BaP concentrations in beef patties cooked for different times using the oven (**A**) or the air fryer (**B**); The relationships between the concentration of BaP and cooking times were evaluated using Spearman’s correlation analysis. The heatmap exhibits the rank correlation coefficients (**C**); all samples were wet samples; asterisks indicate significant differences in BaP concentration between temperatures (* *p* <0.05, ** *p* <0.01, *** *p* <0.001).

When the oven was utilized to cook the beef at 200 °C, BaP concentration in beef was significantly higher at 19 min of cooking compared to 9 min of cooking; the opposite effect was observed when the temperature was set at 140 °C (all *p* < 0.05) (Figure 3A). Spearman’s correlation analysis showed that cooking time and BaP concentration in beef were positively correlated in the 200 °C group, and the rank correlation coefficient was 0.801 (*p* < 0.05) (Figure 3C).

When the air fryer was employed, the BaP concentration in the 19 min group was significantly lower than that in the 9 min group under the temperature of 180 °C or 200 °C, whereas it was higher at 140 °C or 160 °C (all *p* < 0.05) (Figure 3B). Spearman’s correlation analysis showed that cooking time and BaP concentration in beef were positively correlated in the 140 °C (rank correlation coefficients = 0.957) and 160 °C (rank correlation coefficients = 0.965) groups, but they were negatively correlated in the 200 °C (rank correlation coefficients = −0.861) group (all *p* < 0.05) (Figure 3C).

### 3.3. Evaluation of the Effect of Different Cooking Conditions on BaP Concentration in Beef

Linear regression was employed to evaluate the impact of cooking utensils, cooking temperature, cooking time, and oil brushing on the BaP concentration, as depicted in Table 2. In the crude model, after adjusting the above variables separately, we found that the concentration of BaP in beef cooked in the oven was 22.667 (95%CI: 15.984, 29.349) ng/kg higher than that in the air fryer; likewise, the BaP concentration in beef with oil brushing was 38.875 (95%CI: 36.106, 41.644) ng/kg higher than that without oil brushing. Similar results were observed in the multivariate models further adjusted for other variables (4.306 (95%CI: 1.246, 7.365) ng/kg for utensils and 36.722 (95%CI: 33.663, 39.781) ng/kg for oil-brushing). However, the influence of cooking temperature and time on the BaP concentration was not statistically significant in either the crude or multivariate models (both *p* > 0.05).

Stratified analysis was conducted based on cooking utensils, as presented in Table 3. The results showed that the temperature was positively associated with the BaP concentration in the cooked beef when using the oven. With each 20 °C increase in temperature, the BaP concentration increased by 4.456 (95%CI: 3.374, 5.537) ng/kg, regardless of cooking time (0.000 (−1.481, 1.481) ng/kg). However, for beef cooked using the air fryer, neither the cooking temperature nor the cooking time showed a statistically significant association with the BaP concentration (both *p* > 0.05). Interaction analysis indicated a potential interaction between temperature and utensil, influencing the BaP concentration in beef (*p* < 0.001).

## 4. Discussion

This study suggests that cooking beef in an air fryer could result in a lower BaP concentration compared to cooking in an oven, particularly when without oil brushing. The BaP concentration in cooked beef showed a positive correlation with the cooking temperature in the oven, but not in the air fryer. Moreover, the BaP concentration in beef was unaffected by temperature or cooking time when using the air fryer.

According to the report of Sumer et al., the levels of BaP in beef samples during different barbecue cooking could reach up to 0.49 ng/g [27]. Another study also examined the varying concentrations of BaP in beef steak cooked using different types of barbecue (wire and stone) and at various cooking doneness (rare, medium, well-done, and very well-done), with a highest yield up to 0.29 ng/g [28]. In the present study, the BaP concentrations of all analyzed beef patties cooked using both cooking appliances were obviously lower compared with the highest concentrations previously reported during barbecue cooking, and they remained below the standard limit (5 μg/kg) of National Food Safety Standard of China (GB2762-2022) [22]. This is presumably due to the reasonable cooking temperatures and times employed, preventing the patties from burning and thereby reducing BaP formation. In comparison to using the air fryer, beef cooked in the oven exhibited higher BaP concentration, particularly at 200 °C for 19 min, with an increase of 22.34 ng/kg compared to the air fryer. Park et al. revealed that fat dripping onto the heat source is a contributing factor in BaP formation [29]. Because of the structural disparities between the oven and the air fryer, fat from the food is prone to dripping onto the oven’s heat source during cooking, thereby corroborating our experimental findings. Our data suggested that using an air fryer to cook meat should be preferred, particularly when high temperatures are needed.

Heating oils may result in elevated levels of PAHs [30,31]. Air frying, compared to microwave and low-pressure frying, replicates the heat flow akin to boiling oil, dehydrating the food and allowing meat to be cooked with minimal or no additional oil, resulting in significantly reduced fat absorption [1]. Furthermore, the findings of this study revealed that BaP was undetectable in all cooked beef patties prepared without oil brushing, suggesting that oil-free cooking can effectively mitigate BaP concentration. In line with our findings, Huang et al. demonstrated that employing an oil-free cooking method for chicken, pork, and fish resulted in decreased airborne BaP emissions [32]. Apart from preventing the formation of PAH, air frying was also reported to reduce the emitted total volatile organic compounds (VOCs) during the cooking process [33]. The production of acrylamide, a common VOC mainly formed in high-carbohydrate foods, escalates with prolonged cooking conditions of higher temperatures (usually above 120 °C) [12,34]. Nevertheless, the concentration of acrylamide could be reduced by approximately 90% when air frying is utilized instead of traditional deep frying [35]. These findings collectively indicate that the air fryer is capable of effectively reducing the production of food-originated toxic substances and the emission of hazardous fumes during the cooking process owing to oil-free technology.

When cooking in the oven, the cooking temperature is positively correlated with the BaP concentration in beef. Wen et al. demonstrated that the BaP concentration in oven-cooked beef significantly increased with rising temperature [11]. Conversely, when cooking in the air fryer, a negative correlation between cooking temperature and BaP concentration in beef was observed in the 14 min and 19 min groups. Similar findings were reported in the study conducted by Palazoğlu et al. They observed the highest levels of acrylamide in crisps baked at 170 °C, compared to those baked at 180 °C and 190 °C [36]. We speculate that this discrepancy arises from the differing principles underlying the two cooking utensils. Equipped with a fan and a filter plate, the air fryer ensures more uniform distribution of internal temperature [37]. On the other hand, meat droplets produced by the beef can pass through a filter plate. The moisture in the beef is carried away by the air when cooking at high temperatures in an air fryer, allowing the meat droplets to pass through the filter plate, thus resulting in a lower BaP concentration in the beef. Consistently, Lee et al.‘s study showed that the BaP concentration in meat decreased after removing meat droplets [38]. However, the heat distribution in the oven is relatively uneven. As the cooking temperature increases in the oven, the beef’s surface is prone to carbonization and the internal fat decomposition is incomplete, resulting in the production of more BaP. Notably, the BaP concentration increased with cooking time at 140 °C and 160 °C but decreased under 200 °C when using the air fryer. The incomplete fat decomposition and lesser generation of meat droplets due to the low temperature used may render the filter plate less effective, thus leading to this result.

The selection of appropriate cooking methods is crucial for promoting healthy eating habits. Drawing upon the findings regarding the impact of various factors such as cooking utensils, cooking time, cooking temperature, and additional oil brushing on the BaP concentration in beef as revealed by this study, we suggest that BaP concentration in beef is primarily influenced by the choice of cooking utensils and the use of additional oil. Thus, we recommend minimizing the use of cooking oil when preparing beef and selecting appropriate cooking utensils based on the required cooking temperature. This study serves as a guide for individuals to select suitable meat cooking methods to mitigate carcinogen formation and offers foundational data for future research endeavors.

Previous studies have generally suggested that the intake of red meat, like beef, is associated with an increased risk of cardiovascular disease [39,40,41]. Traditional cooking methods such as roasting, pan-frying, and deep-frying may introduce substantial amounts of harmful chemicals into red meat and may further lead to increased health risks [27,28,42]. The evolution of cooking practices has profoundly influenced traditional dietary structures and habits. Thus, the impact of cooking methods has to be taken into account when assessing the association between meat intake and health risks. Together with the results of previous studies, the data from the present study supports the advantages of air fryers in reducing the formation of major hazardous substances such as PAH and VOCs, especially under oil-less cooking conditions [43,44]. Exposure to PAHs and VOCs from food sources has been proven to be related to cardiovascular disease and various cancers, with potential mechanisms involving oxidative stress, inflammatory responses, and genotoxic effects [45,46,47,48]. The application of the air fryer effectively reduces the toxicity of food origin, and thus may promote healthier eating habits and a balanced nutrient supply. However, more elaborate studies are warranted to elucidate the relationship between the consumption of food prepared using an air fryer and possible health risks. Of note, changes in the nutrient content of food after cooking in an air fryer also need to be considered. Ferreira et al. evaluated the essential polyunsaturated fatty acids (PUFAs) in air-fried sardine fillets and found the content of PUFAs significantly degraded in comparison with the control samples [49]. Hence, there is still room for improvement for air fryers to evolve into the optimal cooking method for consumers.

This study also has limitations. We used BaP concentration as a single indicator and did not test for other carcinogens produced during the cooking process, such as heterocyclic aromatic amines (HAAs) and acrylamide. However, the PAHs generated during the cooking process of meat are the main carcinogen contents. It has been reported that the PAHs are higher than HAAs in oven-cooked beef [50,51]. And acrylamide is primarily produced in starch-rich foods [52]. Therefore, the present study used the BaP concentration, the representative carcinogenetic PAHs, in beef to evaluate the quality of the air fryer and the oven. Additionally, the airborne PM_10_ produced during the cooking of chicken was higher in the air fryer compared to the pan [53]. However, emissions of BaP gas during the cooking process were not detected in the present study. Further study was warranted to detect the number of gaseous carcinogens produced/released during meat cooking.

## 5. Conclusions

In this study, we compared the advantages and disadvantages of the air fryer and the oven in terms of BaP concentration in beef and set up subgroups with different cooking times, cooking temperatures, and with or without oil brushing for further analysis and comparison. The results showed that the BaP concentration in beef cooked in the oven was higher than in that cooked in the air fryer. With increasing cooking temperature, the BaP concentration of beef cooked in the oven increased. However, neither cooking temperature nor time had any effect on the BaP concentration of beef cooked in the air fryer. An advantage of the air fryer is that oil-free cooking effectively reduces BaP concentration, thus benefiting human health.

## Figures and Tables

**Table 1:** Effects of different cooking utensils and oil brushing on BaP concentration in beef.

Temperature (°C)	Time (min)	BaP Concentration (ng/kg)			*p* (Oven with Oil Brushing vs. Air Fryer with Oil Brushing)	*p* (Air Fryer with Oil Brushing vs. Air Fryer without Oil Brushing)
		Oven with Oil Brushing	Air Fryer with Oil Brushing	Air Fryer without Oil Brushing		
140	9	38.67 ± 0.58	34.33 ± 0.58	5.00 ± 0.00	<0.001	<0.001
	14	46.33 ± 1.53	48.67 ± 2.52	5.00 ± 0.00	0.242	<0.001
	19	34.33 ± 0.58	59.67 ± 2.31	5.00 ± 0.00	<0.001	<0.001
160	9	45.33 ± 1.16	26.00 ± 0.00	5.00 ± 0.00	<0.001	<0.001
	14	43.00 ± 0.00	38.00 ± 1.00	5.00 ± 0.00	<0.001	<0.001
	19	43.33 ± 1.53	49.33 ± 1.53	5.00 ± 0.00	0.009	<0.001
180	9	46.00 ± 1.00	48.00 ± 1.00	5.00 ± 0.00	0.070	<0.001
	14	48.67 ± 2.52	29.67 ± 0.58	5.00 ± 0.00	<0.001	<0.001
	19	45.67 ± 2.31	43.67 ± 1.16	5.00 ± 0.00	0.251	<0.001
200	9	51.00 ± 2.00	51.33 ± 0.58	5.00 ± 0.00	0.795	<0.001
	14	52.33 ± 3.06	36.67 ± 0.58	5.00 ± 0.00	0.010	<0.001
	19	57.67 ± 1.16	35.33 ± 1.53	5.00 ± 0.00	<0.001	<0.001

*p* < 0.05 was considered as statistically significant. All samples were wet samples.

**Table 2:** Univariate and multivariate analysis of BaP concentration.

Variables	Crude Model	Multivariate Model
Utensils (Air fryer = 0, Oven = 1)	22.667 (15.984, 29.349)	4.306 (1.246, 7.365)
Temperature	0.930 (−2.431, 4.290)	0.930 (−0.187, 2.047)
Time	1.181 (−3.422, 5.783)	1.181 (−0.349, 2.710)
Oil brushing (No = 0, Yes = 1)	38.875 (36.106, 41.644)	36.722 (33.663, 39.781)

**Table 3:** Hierarchical and interaction analyses.

Utensils	Temperature	Time
Oven	4.456 (3.374, 5.537)	0.000 (−1.481, 1.481)
Air fryer	−1.667 (−4.504, 1.171)	3.542 (−0.344, 7.428)
*p* for interaction	<0.001	0.124

## Data Availability

The datasets used and analyzed during the current study are available from the corresponding authors on reasonable request.

## Appendix A

**Table A1:** Number of samples in each experimental group.

Utensils	Oil-Brushing	Time (min)	Temperature( °C)				Total
			140	160	180	200	
Oven	with	9	3	3	3	3	
		14	3	3	3	3	
		19	3	3	3	3	
							36
Air Fryer	with	9	3	3	3	3	
		14	3	3	3	3	
		19	3	3	3	3	
							36
	without	9	3	3	3	3	
		14	3	3	3	3	
		19	3	3	3	3	
							36
